# Field validation of a commercial pasteurization system for heat-treating individual bags of bovine colostrum

**DOI:** 10.3168/jdsc.2026-1081

**Published:** 2026-08-03

**Authors:** Sandra M. Godden, Kenson Boelke, Destiny Smith, Isabela Karels, Robert Schell, Adam Geiger, Whitney A. Knauer

**Affiliations:** 1 Department of Veterinary Population Medicine, University of Minnesota, St. Paul, MN 55108; 2 Department of Dairy and Food Sciences, South Dakota State University, Brookings, SD 57007; 3 CalfStart LLC, Altura, MN 55910; 4 Superior Cattle Solutions, Madison, GA 30650

## Abstract

Our primary objective was to evaluate the effects on IgG and bacteria concentrations of using a commercial pasteurizer system (Matilda Colostrum Pasteurizer and Warmer, DairyTech Inc., Windsor, CO) to heat-treat (HT) individual bags (3.8 L) of bovine colostrum, as compared with treating larger volumes of pooled colostrum (>57 L) in a vat pasteurizer. A secondary objective was to describe concentrations of IgG and bacteria in pre-HT versus post-HT colostrum, whether processed using the Matilda pasteurizer for small volumes or a vat pasteurizer system for large volumes. The study was conducted in summer 2024 on a commercial Midwest Holstein dairy. Each day, high quality (Brix >22%; estimated IgG >50 g/L) colostrum was selected and pooled to create a single unique batch (57–95 L [15–25 gallons; gal]), then transferred into the on-farm 144-L (30 gal) vat pasteurizer. After mixing well and collecting aseptic samples for later testing, 3.8 L were transferred from the pool into a 4-L single-use disposable bag (Perfect Udder [PU] bag system, DairyTech Inc.) which was then placed in a small commercial colostrum pasteurizer system (Matilda Pasteurizer and Warmer, DairyTech Inc.). The large pool and the single bag of colostrum were both then HT in the vat pasteurizer (VAT) and Matilda pasteurizer system (MAT), respectively, at 60°C (140°F) for 60 min. Following rapid cooling to 15.5°C (60°F), aseptic samples of post-HT colostrum were collected from both the VAT and MAT systems and frozen (−20°C) for later analysis. Pre-HT colostrum from the original fresh pool and post-HT colostrum collected from both the VAT and MAT pasteurizer systems were submitted for determination of IgG concentration (g/L) using an radial immunodiffusion assay, as well as aerobic culture to determine total plate count (TPC; cfu/mL) and total coliform count (TCC; cfu/mL). For objective 1, linear regression was used to evaluate if IgG, TPC, or TCC in post-HT colostrum varied by HT method (VAT vs. MAT). For objective 2, after stratifying the data by pasteurization method (VAT or MAT), linear regression was used to evaluate if IgG, TPC, or TCC concentrations were different between the pre- and post-HT samples. Results from 24 unique batches of colostrum showed no effect of HT method on mean (±SE) concentrations of IgG, TPC, or TCC in post-HT colostrum processed using the VAT method (82.7 ± 2.19 g/L; 2.64 ± 0.14 log_10_ cfu/mL; 0.20 ± 0.09 log_10_ cfu/mL, respectively) as compared with the MAT method (82.9 ± 2.19 g/L; 2.96 ± 0.14 log_10_ cfu/mL; 0.07 ± 0.09 log_10_ cfu/mL, respectively). Furthermore, whether processing a larger volume in the VAT system or a small volume in the MAT system, HT resulted in no reduction in IgG, but significantly reduced bacteria levels in post-HT (vs. pre-HT) colostrum. These results show that under the conditions of this study on a single commercial dairy farm, we achieved equally high quality and equally clean colostrum for calves, whether using a vat pasteurizer system for large volumes or using individual bags using the Matilda pasteurizer system for small volumes.

## Graphical Abstract

**Figure:**
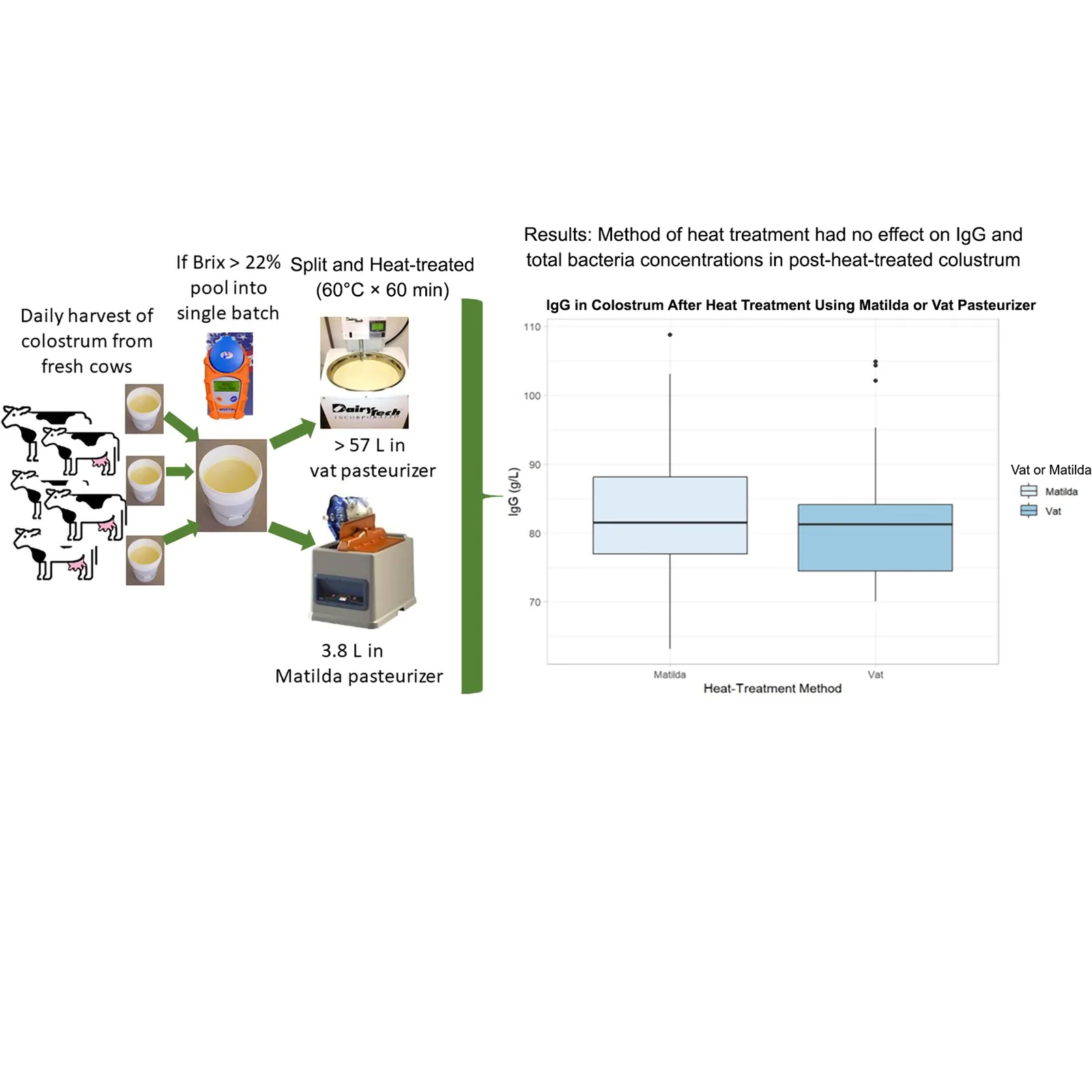

Colostrum management is arguably one of the most important factors in determining dairy calf health and survival, and it has important long-term impacts on rate of gain and future productivity (DeNise et al., 1989; Faber et al., 2005; Urie et al., 2018). Successful colostrum management requires producers to provide calves with a sufficient volume of clean, high-quality colostrum, ideally within the first few hours of life (Godden et al., 2019). Though an important source of nutrients, bioactive compounds, and immune factors, colostrum can also represent one of the earliest potential exposures of dairy calves to infectious agents, including *Mycoplasma* spp., *Mycobacterium avium* ssp. *paratuberculosis*, and *Salmonella* spp. (Streeter et al., 1995; Walz et al., 1997). Furthermore, high concentrations of bacteria in colostrum may interfere with Ig absorption (James et al., 1981). On-farm heat treatment (**HT**) of bovine colostrum using a 60°C × 60 min protocol has been demonstrated to protect important protective IgG while substantially reducing concentrations of bacteria in colostrum (Godden et al., 2006; Donahue et al., 2012). Neonatal calves fed HT colostrum experience improved passive absorption of colostral IgG (Johnson et al., 2007; Elizondo-Salazar and Heinrichs, 2009) and reduced morbidity (Godden et al., 2012; Rafiei et al., 2019) as compared with calves fed fresh (non-HT) colostrum.

As a result of these benefits, the US dairy industry has observed a rapid increase in the adoption of on-farm HT of colostrum, from 0.5% of all operations in 2002 to 5.6% of operations in 2014. In 2014, the highest rate of adoption was observed in medium (100–499 cows; 6.7%) and larger-sized dairies (≥500 cows; 24.0%; USDA-APHIS-VS, 2022). The latter phenomenon is attributable, in part, to the adoption of commercial vat pasteurizer systems (≥10 gallons [**gal**]), also known as batch pasteurizers, wherein the colostrum is poured directly into the barrel of the pasteurizer and then heated. Vat pasteurizers may more easily lend themselves to larger dairies (or possibly dairies adopting seasonal calving systems), which will collect and process moderate-to-large volumes of pooled colostrum daily. Unfortunately, smaller dairies typically will not have a sufficient number of cows calving each day to allow the creation and processing of large pools in a vat pasteurizer system. Furthermore, it may be difficult for smaller dairies to justify the cost of a larger vat pasteurization system. To address these challenges, one previous study demonstrated that producers can successfully HT small volumes by putting colostrum into a single-use disposable bag (e.g., Perfect Udder [**PU**] system, DairyTech Inc., Windsor, CO), then floating the bag in either milk or water in a larger vat pasteurizer system using the 60°C × 60 min HT protocol (Kryzer et al., 2015). However, adopting this floating bag approach to HT single bags of colostrum requires that the farm already have a vat pasteurizer in place, such as for the purpose of feeding a pasteurized whole milk diet to calves.

One solution to the challenge of needing to process small volumes (e.g., 4 L from a single cow) may be the use of a smaller capacity commercial pasteurizer system (Matilda Colostrum Pasteurizer and Warmer, DairyTech Inc., Windsor, CO), which allows producers to either HT or warm 1 or 2 bags (assuming 3-L or 4-L bag capacity) of colostrum at a time. The unit (122 cm long × 87 cm wide × 102 cm high) has 2 connected side-by-side compartments containing a water bath that circulates hot water around the bag for HT or warming cycles, or that circulates cold water around the bag for cooling colostrum. A touch screen allows the operator to select either the HT or warming cycle, and a visual display allows the operator to monitor the machine's status within a given cycle. Time and temperature readings are also displayed and recorded. Additionally, the unit can be operated and monitored using an app on a smartphone.

Although the Matilda pasteurizer offers a convenient approach to HT (and warm) individual bags of colostrum without need for processing larger volumes of pooled colostrum in a vat pasteurizer, the system has never been formally evaluated. Our primary objective was to evaluate the effects on IgG and bacteria concentrations of using a commercial pasteurizer system (Matilda Colostrum Pasteurizer and Warmer) to HT individual bags (3.8-L volumes) of bovine colostrum, as compared with treating larger volumes of colostrum in a vat pasteurizer system. A secondary objective was to describe concentrations of IgG and bacteria in pre-HT versus post-HT colostrum, whether processed using the Matilda pasteurizer for small volumes or a vat pasteurizer system for large volumes. The null hypothesis for objective 1 was that there would be no effect of HT technique (vat vs. individual bag) on IgG and bacteria concentrations in post-HT colostrum. The null hypothesis for objective 2 was that, whether using the vat or the individual bag technique, post-HT colostrum would have similar IgG concentrations but significantly reduced bacteria concentrations as compared with pre-HT colostrum.

This study did not include human or animal subjects and so did not require IACUC/IRB approval. The study was conducted in summer 2024 on a large commercial Holstein dairy farm in the Midwest (∼2,500 milking cows). The farm already routinely heat-treated pooled colostrum daily in a 114-L (30 gal) vat pasteurizer (DairyTech Inc., Windsor, CO). For the purposes of this study, a Matilda Colostrum Pasteurizer and Warmer unit was installed in the same colostrum processing area as the farm's vat pasteurizer. Proper temperature calibration, function, and cleaning of both the Matilda unit and the larger vat pasteurizer were verified once immediately before initiating the study by an experienced equipment specialist and coauthor (RS). All colostrum processing activities were conducted by the study technician and coauthor (KB), with assistance from 2 other study technicians and coauthors (DS and IK).

As part of the farm's normal daily 3× milking routine, parlor staff collected the first- and second-milking colostrum from fresh cows in the parlor at the previous mid-day milking, the previous night milking, and the current day morning milking (∼16 h, 8 h, and 15 min before morning batch assembly, respectively). The colostrum was then refrigerated it in multiple 3- or 5-gallon plastic buckets that had been previously rinsed, manually scrubbed using a brush with hot water and detergent, rinsed, drained, and dried between uses. At the time of batch assembly each morning, which was done immediately after the morning milking of the fresh pen, the study technician (KB) worked alongside herd staff to Brix test all fresh colostrum conserved over the previous 3 milkings. Using a digital refractometer (model PA203X, MISCO), fresh colostrum with a Brix measure >22% was commingled to create a large pool in a vat pasteurizer, with the batch size varying between ∼57 and 95 L (15–25 gal) on any given day. After mixing well, duplicate 10-mL samples of fresh colostrum were hygienically collected (wearing clean disposable gloves) using a sterile single-use pipette into screw-top vials, which were labeled (date, batch, pre-HT) and frozen (−20°C) for later testing. One 3.8-L aliquot of the pooled fresh colostrum mixture was then transferred using a clean, sanitized funnel into a single 4-L PU bag. The PU bag of colostrum was then HT in the Matilda pasteurizer system (**MAT**; 60°C × 60 min), after which it was automatically cooled to 15.5°C (60°F). At the same time, the large pool of colostrum was HT in the vat pasteurizer (**VAT**; 60°C × 60 min), then automatically cooled to 15.5°C (60°F). This rapid (active) cooling, rather than slow (passive) cooling, is recommended to prevent possible damage to colostral immunoglobulins, as well as regrowth of bacteria that could occur if the hot colostrum remains at an elevated temperature for an extended period of time. Study technicians periodically checked the Matilda system throughout each HT cycle by examining the display panel to ensure that the heating up, HT, and cooling down phases of the process were working. Following cooling, duplicate 10-mL samples of post-HT colostrum were aseptically collected into screw top-vials from both the vat pasteurizer and the Matilda PU bag, then labeled (date, batch, post-HT, VAT or MAT) and frozen (−20°C) for later testing.

We originally aimed to collect samples from 27 unique batches of colostrum to provide 80% power and 95% confidence to detect a difference of 5 g/L in colostrum IgG in post-HT colostrum between the VAT and MAT treatments, if a difference existed (assuming an SD of 6.5 g/L; 2-tailed test).

Within 2 mo of collection, all duplicate frozen colostrum samples from pre-HT colostrum (common pool) and post-HT colostrum (VAT and MAT) were transported on ice to the University of Minnesota College of Veterinary Medicine (St. Paul, MN). One sample from each pair was submitted to the University of Minnesota Laboratory for Udder Health (St. Paul, MN) for routine aerobic culture for total plate count (**TPC**; cfu/mL) and total coliform count (**TCC**; cfu/mL). The second (duplicate) frozen colostrum samples from each pair were transported on ice to a local laboratory (Zinpro Corp., Eden Prairie, MN) to undergo testing for colostrum IgG (g/L) using radial immunodiffusion assay analysis, as previously described by Chelack et al. (1993). Laboratory technicians were blinded to the pasteurization technique.

All data were entered into an Excel database file (Microsoft Corp. Redmond, WA) and analyzed using SAS version 9.4 (SAS Institute, Cary, NC). Descriptive statistics were generated to describe characteristics of the IgG (g/L) and Brix (%) measures (mean, SD, range) and for the TPC and TCC concentrations (cfu/mL; median, minimum, quartiles, maximum) for the pre-HT colostrum, as well as for post-HT colostrum processed using either the MAT and VAT HT techniques. However, because of the right-skewed distribution of bacterial concentration data, the TPC and TCC data were log (base 10) transformed before use in analysis of the effect of pasteurization technique. For objective 1, simple linear regression (MIXED procedure) was used to examine the effect of treatment (VAT vs. MAT; explanatory variable) on each of the 3 continuous outcomes of interest in post-HT colostrum samples (IgG [g/L], TPC [log_10_ cfu/mL], and TCC [log_10_ cfu/mL]). For the second objective, after stratifying the dataset by HT method (VAT or MAT), simple linear regression (MIXED procedure in SAS) was used to investigate whether concentrations of IgG, TPC, or TCC (dependent variables of interest) were different in post-HT versus pre-HT samples (explanatory variable of interest). For all models, a *P*-value of ≤0.05 was considered significant.

Ultimately, 28 unique batches of colostrum were assembled and processed over the course of the study. However, there were 4 missing post-HT samples (3 for the MAT group; one for the VAT group), resulting in a complete dataset representing 24 unique batches of colostrum available for the final analysis. The mean (SD; range) IgG and Brix (%) measures for the pooled fresh colostrum were 86.5 (8.9; 72.3–110.5) g/L and 25.1% (0.7%; 23.2%–26.7%), respectively. This high-quality colostrum is typical of what we have observed in other studies of well-managed herds (Godden et al., 2025, unpublished data) and was to be expected, particularly because the herd only selects colostrum with Brix measures >22% to include in the pool. The median (range) TPC and TCC for the pooled fresh colostrum were 9,500 (250–1,300,000) cfu/mL and 845 (40–2,000,000) cfu/mL, respectively (Table 1). After logarithmic transformation, the mean (SD) TPC and TCC concentration in pre-HT colostrum was 4.07 (0.87) log_10_ cfu/mL and 3.21 (1.28) log_10_ cfu/mL respectively. As such, although variation existed, these were relatively clean samples.

**Table 1:** General characteristics of pre- and post-heat-treated (HT) colostrum, processed either in individual bags in a Matilda pasteurizer or in large pooled batches in a vat pasteurizer (n = 24 batches)

Item	Pre-HT Original pooled batch	Post-HT	
		Matilda pasteurizer	Vat pasteurizer
IgG^1^ (g/L)	86.5 (8.9, 72.3–110.5)	82.9 (11.2, 63.2–108.8)	82.7 (10.2, 70.1–104.9)
Brix^1^ (%)	25.1 (0.7, 23.2–26.7)	25.2 (1.0, 23.6–27.9)	25.3 (0.6, 24.4–27.6)
Total plate count^2^ (cfu/mL)	9,500 (250, 3,550, 17,750, 1,300,000)	755 (15, 435, 1,143, 46,000)	480 (35, 114, 963, 15,000)
Total coliform count^2^ (cfu/mL)	845 (40, 299, 3,250, 2,000,000)	0 (0, 0, 0, 50)	0 (0, 0, 0, 210)

1 Values presented as mean (SD; range).

2 Values presented as median (minimum, first quartile, third quartile, maximum).

Results of analysis for objective 1 showed that there was no effect of HT method (VAT vs. MAT) on concentrations of IgG, TPC, or TCC in post-HT colostrum (Table 2). These results show that under the conditions of this study on a single commercial dairy farm, we achieved equally high-quality and equally clean colostrum for calves, whether HT larger volumes using a vat pasteurizer system or small volumes in individual bags using the Matilda pasteurizer system.

**Table 2:** Effect of heat-treated (HT) colostrum in individual bags in a Matilda pasteurizer versus large volumes in a vat pasteurizer on IgG and bacteria concentrations in post-HT bovine colostrum (n = 24 batches)

Parameter^1^	Treatment	Estimate (SE)	Mean (SE)	*P*-value
IgG (g/L)	Matilda	0.17 (3.10)	82.91 (2.19)	0.96
	Vat	Referent	82.74 (2.19)	
TPC (log_10_ cfu/mL)	Matilda	0.32 (0.20)	2.96 (0.14)	0.13
	Vat	Referent	2.64 (0.14)	
TCC (log_10_ cfu/mL)	Matilda	−0.14 (0.13)	0.07 (0.09)	0.30
	Vat	Referent	0.20 (0.09)	

1 TPC = total plate count; TCC = total coliform count.

Equally important is that the results of the analysis for objective 2 showed that HT, whether of a larger volume in the vat pasteurizer or a smaller volume (3.8 L) in an individual bag in the Matilda pasteurizer, preserved important colostrum IgG while causing a significant (*P* < 0.05) reduction in bacteria concentrations in post-HT (vs. pre-HT) colostrum (Table 3). Early attempts to HT or even pasteurize colostrum at higher temperatures (>60°C) generally showed a significant reduction in colostrum IgG (Godden et al., 2003; McMartin et al., 2006). Indeed, a lower temperature (≤60°C) is needed to protect colostral IgG against denaturation (McMartin et al., 2006). The findings of the current study are generally consistent with earlier studies using the 60°C × 60 min HT protocol (Johnson et al., 2007; Donahue et al., 2012; Kryzer et al., 2015; Mann et al., 2020) that reported a small numerical (but usually not statistical) reduction in colostrum IgG. A recent meta-analysis reported that HT at ≤60°C resulted in a 5.38 g/L decrease in colostrum IgG, but noted that this small loss was more than outweighed by the benefits of improved serum IgG (2.65 g/L) and improved serum total protein (+0.21 g/dL) in calves fed HT colostrum (Malik et al., 2022). Although a limitation of our study is that we did not describe transfer of passive immunity in calves fed colostrum HT using the VAT versus MAT systems, several previous studies have documented that lower bacteria concentrations (particularly TPC and TCC) in HT colostrum are associated with enhanced efficiency of absorption of IgG across the small intestine, resulting in increased serum IgG concentrations in the calf (Johnson et al., 2007; Elizondo-Salazar and Heinrichs, 2009; Godden et al., 2012; Kryzer et al., 2015). As a result of reduced pathogen exposure in the gastrointestinal tract and improved transfer of passive immunity (**TPI**), calves fed HT colostrum experience reduced overall morbidity and reduced risk of diarrhea (Godden et al., 2012; Malik et al., 2022). Additionally, an observational study of 306 Holstein calves on one Mexican dairy reported an association between both colostrum quality and cleanliness and calf health, whereby newborn calves fed colostrum with a low TPC (≤100,000 cfu/mL) or a low TCC (≤10,000 cfu/mL) were at reduced risk of pneumonia (Mellado et al., 2017).

**Table 3:** Comparison of IgG and bacteria concentrations in pre- versus post-heat-treated (HT) colostrum, whether heat-treating small volumes in a Matilda pasteurizer or heat-treating large volumes in a vat pasteurizer^1^

Method parameter	Treatment	Estimate (SE)	Mean (SE)	*P*-value
Matilda pasteurizer (3.8 L)				
IgG (g/L)	Post-HT	−3.63 (2.93)	82.91 (2.07)	0.22
	Pre-HT	Referent	86.54 (2.07)	
TPC (log_10_ cfu/mL)	Post-HT	−1.11 (0.23)	2.96 (0.16)	<0.0001
	Pre-HT	Referent	4.07 (0.16)	
TCC (log_10_ cfu/mL)	Post-HT	03.14 (0.27)	0.07 (0.19)	<0.0001
	Pre-HT	Referent	3.21 (0.19)	
Vat pasteurizer (>57 L)				
IgG (g/L)	Post-HT	−3.80 (2.77)	82.74 (1.96)	0.18
	Pre-HT	Referent	86.54 (1.96)	
TPC (log_10_ cfu/mL)	Post-HT	−1.43 (0.23)	2.64 (0.16)	<0.0001
	Pre-HT	Referent	4.07 (0.16)	
TCC (log_10_ cfu/mL)	Post-HT	−3.00 (0.28)	0.21 (0.20)	<0.0001
	Pre-HT	Referent	3.21 (0.20)	

1 TPC = total plate count; TCC = total coliform count.

In past studies, it has been observed that very high quality colostrum (high IgG concentration) is likely more at risk of experiencing damage to IgG as a result of heating, as compared with lower-quality colostrum (Godden et al., 2003). As such, had the colostrum on the study farm been of lower quality, we might expect to observe an even smaller numerical (and nonsignificant) difference in IgG between pre- and post-HT samples, regardless of HT technique. Conversely, had bacteria levels been extremely high (e.g., >1 million cfu/mL) then it is possible that HT might not be as effective in producing a clean (low TPC and low TCC) finished product. However, we have no reason to believe that this impact would be different when comparing efficacy between the 2 HT techniques.

This is not to say that HT is the only approach to providing clean colostrum to calves. Producers should always do their best to reduce bacterial contamination through cleaning and disinfection of the udder before harvesting colostrum, as well as through proper cleaning and sanitation of all colostrum milking, storage, or feeding equipment. Briefly, an example of recommended steps for cleaning and sanitizing milking, storage, or feeding equipment would be (1) rinsing in lukewarm water (∼32°C), (2) manually washing with a brush for 2 to 3 min using hot water (≥60°C) mixed with chlorinated alkaline detergent, (3) rinsing with cold water, (4) rinsing a second time with cold water mixed with 2 to 3 pH acid and a 50 mg/kg solution of chlorine dioxide, and (5) drying (Bechtel, 2017).

Also important is appropriate handling of colostrum so as to reduce bacterial proliferation in stored colostrum, such as through rapid refrigeration or freezing (Bey et al., 2007; Stewart et al., 2005; Trudeau et al., 2025) or the use of approved preservatives such as potassium sorbate (Godden et al., 2025; Denholm et al., 2026). Given that USDA-licensed colostrum replacement products undergo pasteurization, spray drying, and subsequent testing to ensure they are free of pathogens, feeding a sufficient dose of a high-quality USDA-licensed colostrum replacement product can also be an effective means of reducing the risk exposure to certain pathogens that may be shed in colostrum by the dam, while also ensuring high levels of TPI (Godden et al., 2009; Lago et al., 2018; da Silva et al., 2020). However, HT of colostrum can be a very beneficial management strategy to further reduce the risk for bacterial contamination of colostrum, particularly for operations that have challenges in preventing contamination during colostrum harvest or storage, as well as for farms with endemic diseases in the adult herd that may be transmitted through colostrum (Streeter et al., 1995; Walz et al., 1997). For example, the 60°C × 60 min HT protocol for colostrum can eliminate viable *Mycoplasma bovis*, *Listeria monocytogenes*, *Escherichia. coli* O157:H7, and *Salmonella enteritidis*, as well as significantly reduce the risk for viable *Mycobacterium avium* ssp. *paratuberculosis* (Godden et al., 2006).

Although the colostrum HT protocol used in this study (60°C × 60 min) has been previously validated, this protocol was originally developed, and later validated, using a vat (or batch) pasteurization technique (Godden et al., 2006; Donahue et al., 2012). With the exception of one earlier study that successfully HT individual bags of colostrum by floating them in water in a vat pasteurizer (Kryzer et al., 2015), studies have been previously lacking to evaluate commercial pasteurizer systems designed specifically to HT individual bags of colostrum. This is the first formal study to evaluate the effects on colostrum IgG and bacteria concentrations of using a commercial pasteurizer system (Matilda Colostrum Pasteurizer and Warmer, DairyTech Inc., Windsor, CO) to HT individual bags of bovine colostrum. Several years of research plus field experience has taught us that successful HT of colostrum requires (1) precise control of the HT temperature (60°C × 60 min) without allowing large temperature fluctuations (i.e., not to exceed 61°C); (2) rapid and active heating and cooling before and after the HT phase, respectively, to prevent IgG damage that might occur with prolonged times spent at higher temperatures; and (3) constant agitation throughout the entire process to achieve more rapid and even heating (and cooling) of the contents of the bag or vat. Although the current study showed that colostrum was successfully HT using the Matilda pasteurizer, we have field experience (unpublished) with a different commercial pasteurization system in which HT failure occurred because the system was unable to precisely control the HT temperature or the system was unable to rapidly cool the colostrum when processed in individual bags. Thus, the results of previous vat pasteurizer studies and of the current study should not automatically be transferred to other commercial systems that process colostrum in individual bags. It is important that different commercial pasteurization systems be independently evaluated in order to protect dairy producers and the animals they care for.

In summary, under the conditions of this study on a single commercial dairy farm, and using high quality colostrum, there was no difference between post-HT IgG concentration or bacteria concentrations (TPC and TCC) when comparing colostrum HT in larger volumes using a vat pasteurizer system or small volumes in individual bags using the Matilda pasteurizer system. Furthermore, heat treatment, whether of a large volume in the vat pasteurizer, or an individual bag in the Matilda pasteurizer, preserved colostrum IgG while also significantly reducing bacteria levels in post-HT (vs. pre-HT) colostrum. A strength of this study was that it was conducted on a large commercial dairy and using commercially available pasteurization equipment, though it is a limitation that we only evaluated the system on one farm. Future research should explore if the results are repeatable on other farms and under different conditions. Another possible limitation of this study is that it was only conducted using a single vat pasteurizer and a single Matilda pasteurizer system. Nonetheless, both systems were inspected to verify proper temperature calibration and function before study start. Also, study technicians periodically checked the Matilda system throughout each HT cycle by examining the display panel to ensure that the heating up, HT, and cooling down phases of the process were working.

The results of this study should not be extrapolated to other commercial colostrum pasteurizer systems that process small volumes (e.g., individual bags) of colostrum, given that differences in equipment design, function, and both the accuracy and precision of temperature control and cooling can affect results. As such, other commercial systems that are designed to HT individual bags of colostrum should be independently evaluated. Furthermore, and regardless of the colostrum (or milk) pasteurizer system in place on a given farm, producers are strongly encouraged to have equipment regularly inspected by a local support expert to ensure proper temperature calibration, functioning, and cleaning and sanitation. Though an exact frequency has not been established, theoretically this could be done monthly, similar to the frequency of evaluating milking parlor equipment settings and function.

## References

[bib1] Bechtel W. (2017). Calf Facilities: Clean, Clean, Clean!. Dairy Herd Management.

[bib2] Bey R., Godden S., Lillegaard H., Stewart S., Rapnicki P., Farnsworth R. (2007). Proc. Annu. Meet. Minnesota Dairy Health Management Conference.

[bib3] Chelack B.J., Morley P.S., Haines D.M. (1993). Evaluation of methods for dehydration of bovine colostrum for total replacement of normal colostrum in calves. Can. Vet. J..

[bib4] da Silva A.P., Faria de Toledo A., Cezar A.M., Coelho M.G., Virgínío G.F., Poczynek M., Silva M.D., Haines D.M., Campos M., Bittar C.M.M. (2020). Passive transfer and neonatal health in dairy calves receiving maternal colostrum and/or a colostrum replacer. Livest. Sci..

[bib5] Denholm K.S.M., Carvalho M., Haggerty A., Jones R. (2026). Effects of various concentrations and volumes of potassium sorbate on colostrum bacterial contamination over time at ambient and refrigeration temperatures. J. Dairy Sci..

[bib6] DeNise S.K., Robison J.D., Stott G.H., Armstrong D.V. (1989). Effects of passive immunity on subsequent production in dairy heifers. J. Dairy Sci..

[bib7] Donahue M., Godden S.M., Bey R., Wells S., Oakes J.M., Sreevatsan S., Stabel J., Fetrow J. (2012). Heat treatment of colostrum on commercial dairy farms decreases colostrum microbial counts while maintaining colostrum immunoglobulin G concentrations. J. Dairy Sci..

[bib8] Elizondo-Salazar J.A., Heinrichs A.J. (2009). Feeding heat-treated colostrum to neonatal dairy heifers: Effects on growth characteristics and blood parameters. J. Dairy Sci..

[bib9] Faber S.N., Faber N.E., McCauley T.C., Ax R.L. (2005). Effects of colostrum ingestion on lactational performance. Prof. Anim. Sci..

[bib10] Godden S.M., Lombard J.E., Woolums A.R. (2019). Colostrum management for dairy calves. Vet. Clin. North Am. Food Anim. Pract..

[bib11] Godden S., McMartin S., Feirtag J., Stabel J., Bey R., Goyal S., Metzger L., Fetrow J., Wells S., Chester-Jones H. (2006). Heat-treatment of bovine colostrum II: Effects of heating duration on pathogen viability and immunoglobulin G. J. Dairy Sci..

[bib12] Godden S.M., Smith S., Feirtag J.M., Green L.R., Wells S.J., Fetrow J.P. (2003). Effect of on-farm commercial batch pasteurization of colostrum on colostrum and serum immunoglobulin concentrations in dairy calves. J. Dairy Sci..

[bib13] Godden S.M., Haines D.M., Hagman D. (2009). Improving passive transfer of immunoglobulins in calves I: Dose effect of feeding a commercial colostrum replacer. J. Dairy Sci..

[bib14] Godden S.M., Royster E., Timmerman J., Crooker B.A., Brown N. (2025). Efficacy of a stabilized liquid potassium sorbate preservative to prevent bacterial proliferation in bovine colostrum and milk. J. Dairy Sci..

[bib15] Godden S.M., Smolenski D.J., Donahue M., Oakes J.M., Bey R., Wells S., Sreevatsan S., Stabel J., Fetrow J. (2012). Heat-treated colostrum and reduced morbidity in preweaned dairy calves: Results of a randomized trial and examination of mechanisms of effectiveness. J. Dairy Sci..

[bib16] James R.E., Polan C.E., Cummins K.A. (1981). Influence of administered indigenous microorganisms on uptake of [iodine-125] gamma-globulin in vivo by intestinal segments of neonatal calves. J. Dairy Sci..

[bib17] Johnson J.L., Godden S.M., Molitor T., Ames T., Hagman D. (2007). The effect of feeding heat treated colostrum on passive transfer of immune and nutritional parameters in dairy calves. J. Dairy Sci..

[bib18] Kryzer A.A., Godden S.M., Schell R. (2015). Heat-treated (in single aliquot or batch) colostrum outperforms non-heat-treated colostrum in terms of quality and transfer of immunoglobulin G in neonatal Jersey calves. J. Dairy Sci..

[bib19] Lago A., Socha M., Geiger A., Cook D., Silva-del-Río N., Blanc C., Quesnell R., Leonardi C. (2018). Efficacy of colostrum replacer versus maternal colostrum on immunological status, health, and growth of preweaned dairy calves. J. Dairy Sci..

[bib20] Malik M.I., Rashid M.A., Raboisson D. (2022). Heat treatment of colostrum at 60°C decreases colostrum immunoglobulins but increases serum immunoglobulins and serum total protein: A meta-analysis. J. Dairy Sci..

[bib21] Mann S., Curone G., Chandler T.L., Moroni P., Cha J., Bhawal R., Zhang S. (2020). Heat treatment of bovine colostrum: I. Effects on bacterial and somatic cell counts, immunoglobulin, insulin, and IGF-I concentrations, as well as the colostrum proteome. J. Dairy Sci..

[bib22] McMartin S., Godden S., Metzger L., Feirtag J., Bey R., Stabel J., Goyal S., Fetrow J., Wells S., Chester-Jones H. (2006). Heat treatment of bovine colostrum. I: Effects of temperature on viscosity and immunoglobulin G level. J. Dairy Sci..

[bib23] Mellado M., Torres E., Veliz F.G., de Santiago A., Macias-Cruz U., Garcia J.E. (2017). Effect of quality of colostrum on health, growth and immunoglobulin G concentration in Holstein calves in a hot environment. Anim. Sci. J..

[bib24] Rafiei M., Ghoorchi T., Toghdory A., Moazeni M., Khalili M. (2019). Effect of feeding heat-treated and unheated colostrum on immunoglobulin G absorption, health and performance of neonatal Holstein dairy calves. Acta Scientiarum.

[bib25] Stewart S., Godden S., Bey R., Rapnicki P., Fetrow J., Farnsworth R., Scanlon M., Arnold Y., Clow L., Mueller K., Ferrouillet C. (2005). Preventing bacterial contamination and proliferation during the harvest, storage and feeding of fresh bovine colostrum. J. Dairy Sci..

[bib26] Streeter R.N., Hoffsis G.F., Bech-Nielsen S., Shulaw W.P., Rings D.M. (1995). Isolation of *Mycobacterium paratuberculosis* from colostrum and milk of subclinically infected cows. Am. J. Vet. Res..

[bib27] Trudeau S., Bourel-Conroy C., Hérambert P., Guatteo R., Buczinski S. (2025). Monitoring colostrum harvesting and distribution equipment cleanliness with adenosine triphosphate luminometry before and after recommendations to improve hygiene practices in French dairy farms. J. Dairy Sci..

[bib28] Urie N.J., Lombard J.E., Shivley C.B., Kopral C.A., Adams A.E., Earleywine T.J., Olson J.D., Garry F.B. (2018). Preweaned heifer management on US dairy operations: Part V. Factors associated with morbidity and mortality in preweaned dairy heifer calves. J. Dairy Sci..

[bib29] USDA-APHIS-VS (2022). https://www.aphis.usda.gov/sites/default/files/dairy-trends-hlth-mngmnt-1991-2014.pdf.

[bib30] Walz P.H., Mullaney T.P., Render J.A., Walker R.D., Mosser T., Baker J.C. (1997). Otitis media in preweaned Holstein dairy calves in Michigan due to *Mycoplasma bovis.*. J. Vet. Diagn. Invest..

